# Profiling of phenolic compounds and their antioxidant and anticancer activities in pandan (*Pandanus amaryllifolius* Roxb.) extracts from different locations of Malaysia

**DOI:** 10.1186/1472-6882-13-341

**Published:** 2013-12-01

**Authors:** Ali Ghasemzadeh, Hawa ZE Jaafar

**Affiliations:** 1 Department of Crop Science, Faculty of Agriculture, University Putra Malaysia, 43400 Serdang, Selangor, Malaysia

**Keywords:** *Pandanus amaryllifolius*, Flavonoids, HPLC, Antioxidant activity, DPPH, FRAP, Anticancer activity, MCF-7

## Abstract

**Background:**

Phytochemicals and antioxidants from plant sources are of increasing interest to consumers because of their roles in the maintenance of human health. Most of the secondary metabolites of herbs are used in a number of pharmaceutical products.

**Methods:**

Secondary metabolites composition and content of five flavonoids and three phenolic acids were evaluated and determined in *Pandanus amaryllifolius* extracts from three different locations of Malaysia by RP-HPLC; Total phenolic and total flavonoid content were determined using Folin-Ciocalteau and aluminum chloride colorimetric assay; The antioxidant activity of the extracts was determined by the ferric reducing antioxidant potential (FRAP) assay and 1,1-diphenyl-2-picrylhydrazyl (DPPH) assays. MTT (3-(4,5-Dimethylthiazol-2-yl)-2,5-diphenyltetrazolium bromide) Assay was employed to screen anticancer activity of extracts against MCF-7 cancer cell line.

**Results:**

Highest value of total flavonoids (TF) and total phenolics (TP) was observed in pandan extract from Bachok locattion (1.87 mg/g DW and 6.72 mg/g DW) followed by Klang (1.32 mg/g DW; 5.07 mg/g DW) and Pontian (1.12 mg/g DW; 4.88 mg/g DW). Rutin just detected from Bachok location with value of 0.082 mg/g DW. High content of epicatechin (0.035 mg/g DW) and naringin (0.325 mg/g DW) were observed from Bachok location while, highest content of catechin (0.613 mg/g DW) and kaempferol (0.278 mg/g DW) was observed in pandan extract from Klang location. The extract of pandan from Bachok exhibited highest value of gallic acid (0.423 mg/g DW) and cinnamic acid (0.084 mg/g DW). Ferrulic acid just detected from pandan extract of Bachok location with concentration of 0.281mg/g DW. Between studied locations Bachok exhibited highest value of DPPH (64.27%) and FRAP (517.2 μm of Fe (II)/g) activity followed by Klang (52.16%; 448.6 μm of Fe (II)/g) and Pontian (50.10%; 314.8 μm of Fe (II)/g). The preliminary screening showed pandan extracts from 3 locations possessed anticancer promoting activity against MCF-7 cell line, with 78.3%, 70.5% and 67.4% inhibition rate, respectively. Maximum MCF-7cell line inhibition was observed in pandan extract from Bachok location.

**Conclusions:**

The samples collected from the North (Bachok) exhibited the highest TP, TF antioxidant and anticancer activity while those from the Southern portion (Pontian) appeared to have the lowest content of TP, TF and antioxidant activity.

## Background

Most plants are major sources of natural products used in pharmaceuticals, agrochemicals, flavour and fragrance ingredients, food additives, and pesticides. Secondary compounds are unique to a species or group, and they are important for defense, protection and competition [1]. Most of these compounds are commonly used as flavourings, medicines, or recreational drugs. Secondary chemicals are important in plant use by humans. Most pharmaceuticals are based on plant component structures, and secondary metabolites are widely used especially in Asia [2]. Phenolic compounds are famous group of secondary metabolites with wide pharmacological activities. Flavonoids are an important group of secondary metabolites and are a source of bioactive compounds in plants [3]. They are also a kind of natural product with antioxidant properties capable of scavenging free superoxide radicals, having anti-aging properties as well as reducing the risk of cancer. Park et al. [4] showed that some flavonoid components in green tea are effective in inhibiting cancer or induce mechanisms that may kill cancer cells and inhibit tumor invasion. It was found that flavonoids reduced blood-lipids and glucose, and enhanced human immunity [5,6]. The effect of flavonoids on human health is the result of their ability to induce human protective enzyme systems [7]. Several studies have suggested that flavonoids such as catechin and rutin are able to control cancer cell growth in the human body [8-10].

Pandan (*Pandanus amaryllifolius* Roxb.) is a tropical plant of the family Pandanaceae in the screw pine genus. Pandan leaf, often known as screw pine, because they resemble the pineapple with the spiral arrangement of long, narrow and strap-shaped green leaves [11]. Even though the Pandanaceae family comprises approximately 600 species, there are only *Pandanus amaryllifolius* Roxb. *and Pandanus odoratissimus* Linn. that have fragrant leaves and flowers, respectively [12]. The genus Pandanus from the family Pandanaceae comprises approximately 600 species that are widely distributed in tropical and subtropical regions. The sweet and delightful flavour of pandan leaves, which is well-known as a source of natural flavouring, is widely used in various parts of South-East Asian countries including India, Thailand, Indonesia and Malaysia. For example, pandan leaves are commonly used when preparing rice dishes as a means of enhancing flavour. In addition, pandan leaves are also used in making other food, such as desserts, sweets, coconut jam and ice cream. Due to the high chlorophyll content, pandan leaves are also a popular green colourant for food [13]. In Malaysia, herbs and spices are generally eaten raw and fresh as vegetable (salad), especially among the Malay community. Most of these herbs and spice are believed to be associated with high antioxidant activities and have many benefits on human body. A systematic search for anticancer plants began in the middle of the 21^th^ century through the applications of appropriate biological screening assays. The chemopreventive properties of plants extracts are often investigated via screening against a panel of human cancer cell lines. In recent years, there has been increasing interest in organically grown food because people believe that they have less pesticide residues and are healthier [14]. However, most studies on this subject have reported conflicting results and it is unclear whether or not organically grown food contains more health-promoting phytochemicals as opposed to conventionally grown food. Information about flavonoid compounds of Malaysian pandan and their antioxidant and anticancer activity are still scarce and some information and such data would be useful to provide information on foods containing high levels of beneficial components. The present investigation was undertaken to screen phytochemical potential and their antioxidant activities in *P. amaryllifolius* collected from three different location of Malaysia. In addition *in vitro* anticancer properties of the extracts against breast cancer cell lines were also investigated.

## Methods

### Plant material and maintenance

Fresh leaves of *P. amaryllifolius* were collected locally from three different province of Malaysia namely: Johor (pontian, south), Selangor (Klang, Central) and Kelantan (Bachok, North). The samples were identified by Malaysian Agriculture Research and Development Institute (MARDI). Voucher specimens of *P. amaryllifolius* Kelantan (MTP008/1), Selangor (MTP008/2) and Johor (MTP008/3) were collected. Malaysian Agriculture Research and Development Institute (MARDI) verified and kept samples. The leaves were shade dried and were powdered using mechanical grinder. This powered material is used for further analysis.

### Preparation of flavonoids extract

Leaf samples (0.25 g) were extracted with 20 mL of methanol on a shaker for 2 h at room temperature. The extract solution was treated with 6 M HCl (5 ml) and refluxed at 90°C for 2 h. The hydrolysed sample was cooled to room temperature and filtered through a 0.45 μm membrane [15].

### Determination of total flavonoids

The TF were measured following a previously reported spectrophotometric method [16]. Briefly, extracts of each plant material (1 mL) were diluted with 4 mL water in a 10 mL volumetric flask. Initially, 5% NaNO_2_ solution (0.3 mL) was added to each volumetric flask; after 5 min, 10% AlCl_3_ (w/v) was added; and at 6 min, 1.0 M NaOH (2 mL) was added. Absorbance of the reaction mixture was read at 430 nm.

### Separation and analysis of flavonoids by HPLC

Reversed-phase HPLC was used to assay flavonoid composition. The Agilent HPLC system used consisted of a Model 1100 pump equipped with a multi-solvent delivery system, an L-7400 ultraviolet (UV) detector, and fitted with an Agilent C18 (5 μm, 4.6 × 250 mm) column. The mobile phase consisted of: (A) 2% acetic acid (CH_3_COOH) and (B) 0.5% acetic acid-acetonitrile (CH_3_CN), (50:50 v/v). The mobile phase was filtered under vacuum through a 0.45 um membrane filter before use. Gradient elution was performed as follows: 0 min, 95:5; 10 min, 90:10; 40 min, 60:40, 55 min, 45:55; 60 min, 20:80; and 65 min, 0:100. The flow rate was maintained at 1 mL/min and UV absorbance was measured at 260–360 nm. The operating temperature was maintained at room temperature [17]. Identification of the flavonoids was achieved by comparison of retention times with standards, UV spectra and UV absorbance ratios after co-injection of samples and standards. The standards [(+)-Catechin, (-)-Epicatechin, Naringin, Rutin and Kaempferol] were purchased from Sigma–Aldrich (St. Louis, MO, USA).

### Preparation of phenolic acids extract

Phenolics extracts were prepared by first carefully pipetting phosphoric acid (H_3_PO_4_, 1.2 mL) into about 950 mL water in a 1 L volumetric flask, mixing and bringing to volume with water. Then leaves (0.25 g) were extracted with 20 mL, of this phosphoric acid solution. Five mL of 6 M HC1 was added to each extract to give a 25 mL solution of 1.2 M HC1 in 50% MeOH. Extracts were refluxed at 90°C for 2 h and solution was filtered through a 0.45 μm filter [18].

### Determination of total phenolic content

The total phenolic content was determined following the method of Kim et al. [19]. Briefly, 1 mL of extract was added to deionized water (10 mL) and Folin–Ciocalteu phenol reagents (1.0 mL). After 5 min, 20% sodium carbonate (2.0 mL) was added to the mixture. The solution was kept in total darkness, and the absorbance was measured at 750 nm using a spectrophotometer (U-2001, Hitachi Instruments Inc., Tokyo, Japan).

### Separation and analysis of phenolic acids by HPLC

An Agilent HPLC system (Tokyo, Japan) consisting of a Model 1100 pump equipped with a multisolvent delivery system and a L-7400 ultraviolet (UV) detector was used. The column was an Agilent C18 (5 μm, 4.6 × 250 mm). The mobile phase was composed of phosphoric acid (aqueous) and (B) acetonitrile and gradient elution was performed as follows: 0 min, 85:15; 12 min, 75:25; 20 min, 75:25; 22 min, 85:15 and 30 min, 85:15. The mobile phase was filtered under vacuum through a 0.45 lm membrane filter before use. The flow rate and injection volume were 1 mL/min and 20 μL. UV absorbance was measured at 220–360 nm. The operating temperature was maintained at room temperature [18]. Identification of the phenolic acids were achieved by comparison with retention times of standards, UV spectra and calculation of UV absorbance ratios after co-injection of samples and standards. Commercial standards [Gallic acid, trans-Cinnamic acid and trans-Ferulic acid] were purchased from Sigma–Aldrich.

### Determination of antioxidant activity

#### Ferric reducing antioxidant potential (FRAP) assay

The stock solutions consisted of 300 mM acetate buffer, 10 mM TPTZ (2,4,6-tripyridyl-S-triazine) solution in 40 mM HCl, and 20 mM FeCl_3_ solution. Acetate buffer (25 mL) and TPTZ (2.5 mL) were mixed, and 2.5 mL FeCl_3_ added. Leaf extract (150 μL) was added to 2850 μL of the FRAP solution and kept for 30 min in the dark place. The absorbance of solution was measured at 593 nm using a spectrophotometer (U-2001, Hitachi Instruments Inc., Tokyo, Japan) [20].

### 1,1-Diphenyl-2-picrylhydrazyl (DPPH) assay

1,1-Diphenyl-2-picrylhydrazyl (DPPH) was purchased from Sigma–Aldrich. Butylated hydroxytoluene (BHT) and α-tocopherol were purchased from Merck. The radical scavenging ability was determined using the method described in Mensor et al. [21]. Briefly, an alcohol solution of DPPH (1 mL, 3 mg/mL) was added to 2.5 mL samples containing different concentrations of extracts. The samples were first kept in the dark at room temperature and their absorbance was read at 518 nm after 30 min. The antiradical activity was determined using the following formula:

$$\begin{array}{l} \mathrm{Percent}\;\left(\%\right)\;\mathrm{inhibition}\;\mathrm{of}\;\mathrm{DPPH}\;\mathrm{activity}\\ \;=\left[\left(A_{0}-A_{1}\right)/A_{0}\right]\times 100\% \end{array}$$

Where A_0_ is the absorbance value of the blank sample or control reaction, and A_1_ is the absorbance value of the test sample. The optic density of the samples and controls were measured in comparison to ethanol. BHT (butylhydroxytoluene) and Vit C, were used as positive controls.

### Determination of anticancer activity

#### Cell culture and treatment

Human breast carcinoma (MCF-7) and normal (MCF-10A) cells were cultured in 100 μL of RPMI 1640 media (Roswell Park Memorial Institute) containing 10% fetal bovine serum (FBS). MCF-7 cells were incubated overnight at 37°C in 5% CO_2_ for cell attachment.

#### MTT (3-(4,5-Dimethylthiazol-2-yl)-2,5-diphenyltetrazolium bromide) assay

The assay was conducted as follows: Cancer cells were seeded in 96-well plates at a density of 1 × 104 cells/well in 100 μL RPMI. At 24 h after seeding, the medium was removed and the cells were incubated for 3 days with RPMI in the absence or presence of various concentrations of extracts. Extract concentrations used ranged from 20, 40, 80, 160, 320 and 640 μg/mL. After incubation, 20 μL of MTT [3-(4,5-dimethylthiazol-2-yl)-2,5-diphenyltetrazolium bromide] reagent was added into each well. The plate was incubated again for 4 h in a CO_2_ incubator at 37°C. The resulting MTT–products were determined by measuring the absorbance at 570 nm using ELISA reader [22]. Each point represents the mean of triplicate experiments. The cell viability was determined using the formula:

$$\begin{array}{l} \mathrm{Viability}\;\left(\%\right)=\left(\mathrm{optical}\;\mathrm{density}\;\mathrm{of}\;\mathrm{sample}/\mathrm{optical}\;\mathrm{density}\;\mathrm{of}\;\mathrm{control}\right)\\ \;\times 100 \end{array}$$

### Statistical analysis

All analytical values shown represent the means of three replicates. Data were analysed using analysis of variance by Statistical Analysis System (SAS 9.0). Mean separation test between treatments was performed using Duncan multiple range test and a P-value ≤ 0.05 was regarded as significant.

## Results and discussion

### The concentrations of TF and some flavonoid compounds

The results obtained from the preliminary analysis of flavonoid compounds are shown in Table 1. There was a significant difference between the three locations for TF production in pandan. Highest value of TF content in pandan was observed in Bachok (1.87 ± 0.246 mg/g DW) location followed by klang (1.32 ± 0.211 mg/g DW) and pontian (1.12 ± 0.177 mg/g DW). In this research, 5 flavonoid compounds were detected and identified from pandan extract. There was a significant difference (P ≤ 0.05) between the three locations for TF content.

**Table 1:** The concentrations of TF and some flavonoid compounds detected in pandan extracts from three different locations

	**Bachok**	**Klang**	**Pontian**
TF	1.87 ± 0.246^a^	1.32 ± 0.211^b^	1.12 ± 0.177^c^
Rutin	0.082 ± 0.028^a^	ND	ND
Epicatechin	0.035 ± 0.045^a^	0.022 ± 0.039^a^	0.008 ± 0.042^a^
Catechin	0.527 ± 0.024^b^	0.613 ± 0.015^a^	0.153 ± 0.046^c^
Kaempferol	0.158 ± 0.033^b^	0.278 ± 0.029^a^	ND
Naringin	0.325 ± 0.025^a^	0.223 ± 0.026^b^	ND

All analyses are the mean of triplicate measurements ± standard deviation. Results expressed in mg/g DW. a,b,c represents Duncan multiple range test letters. Means not sharing a common letter were significantly different at P ≤ 0.05. ND: not detected.

As shown in Table 1, rutin detected just from one locations (Bachok) and high value of this flavonoid was recorded 0.082 ± 0.028 mg/g DW. Bachok location also, showed high epicatechin content (0.035 ± 0.045 mg/g DW) compared to Klang location but, there were no significant differences between locations for epicatechin content in pandan extracts. High concentration of catechin was observed in Klang location (0.613 ± 0.015 mg/g DW).

Kaempferol is a rare flavonoid component in plants, but it was detected in the pandan extracts just from two locations (Bachok and Klang) with remarkable concentration. Klang showed highest concentration of kaempferol (0.278 ± 0.029 mg/g DW) followed by Bachok (0.158 ± 0.033 mg/g DW). These kaempferol contents were higher than those recorded in pegaga (0.0205 mg/g DW), sengkuang (0.037 mg/g DW), carrot (0.140 mg/g DW), green chilli (0.039 mg/g DW) and white radish (0.0383 mg/g DW) [23]. In other study, Tolonen et al. [24] identified kaempferol in white cabbages with concentration of 0.9 mg/kg FW, and it was the only flavonoid found. Meanwhile, Kim and Lee [25] detected about 0.1–0.8 mg/g FW of kaempferol contents in green cabbages. In current study kaempferol was not detected in pandan extract from pontian location.

**Figure 1:**
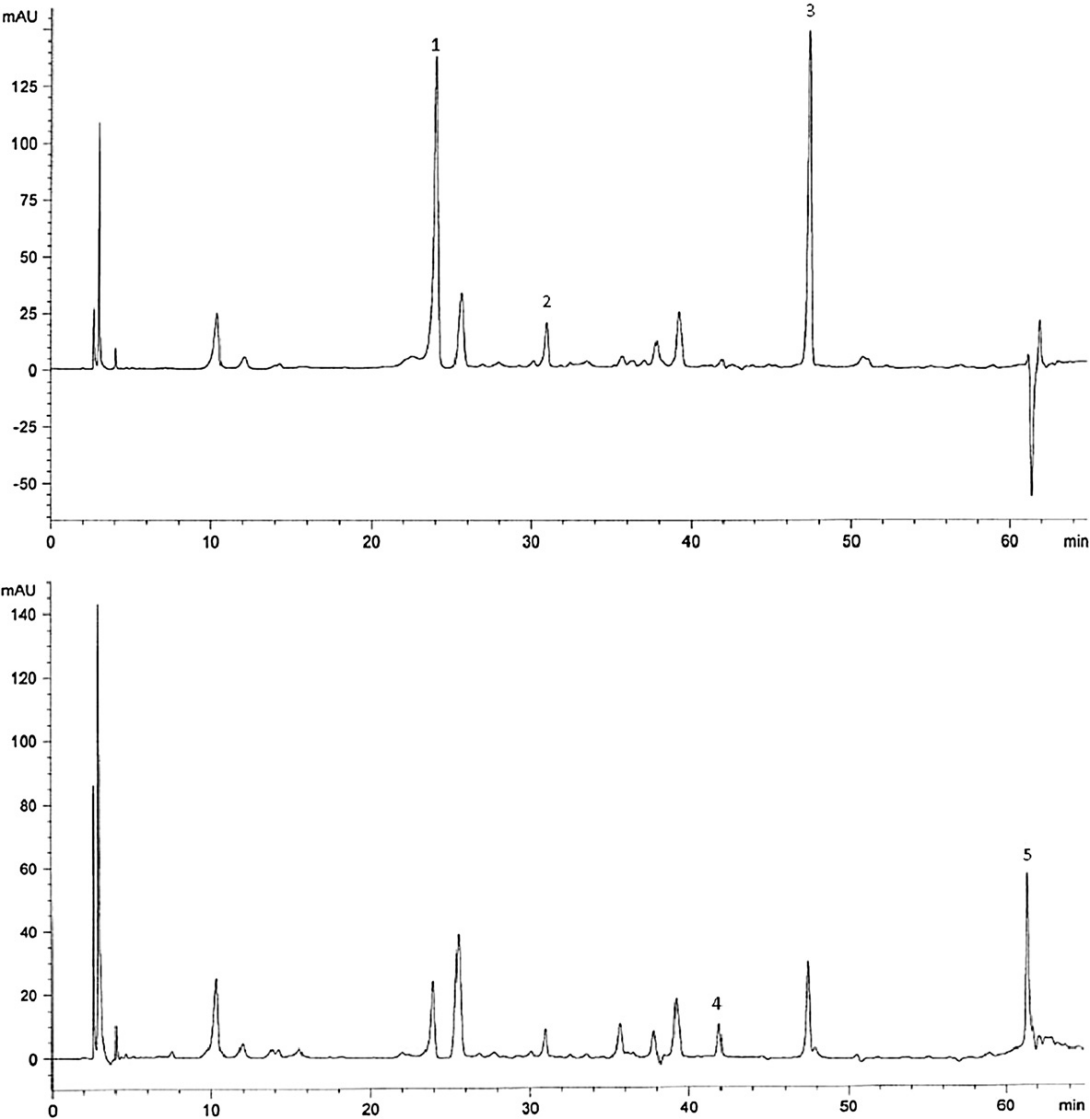
**HPLC chromatograms of flavonoid compounds extracted from pandan, Bachok location.** The identified peaks are: catechin (1), epicatechin (2), naringin (3), rutin (4) and kaempferol (5).

Naringin was also detected from pandan extract with substantial concentration. Between studied locations Bachok showed highest value 0.325 ± 0.025 mg/g DW followed by Klang 0.223 ± 0.026 mg/g DW. Naringin was not detected from pandan in Pontian location. Zhang et al. [26] identified rutin in pandan (location of Felorida, USA) with concentration of 0.356 mg/100 g DW. Generally, between identified flavonoid compounds the important compound based on concentration from high to low were: catechin > naringin > kaempferol > rutin > epicatechin. Results imply that catechin is abundant flavonoid compounds in pandan. Figure 1 shows the HPLC chromatogram of pandan extracts from Bachok location.

### The concentrations of TP and some phenolic acids

Usually, phenolics that possess antioxidant activity are known to be mainly flavonoids and phenolic acids. Phenolic acids are a major class of phenolic compounds, widely occurring in the plant kingdom especially in herbs and vegetables. As shown in Table 2, pandan extract from Bachok present highest content of TP (6.72 ± 0.355 mg/g DW) followed by Klang (5.07 ± 0.406 mg/g DW) and Pontian (4.88 ± 0.477 mg/g DW). A significant difference (P ≤ 0.05) was observed between Bachok and Klang in TP content but, differences between Klang and Pontian was not significant. However, compared to some of potent herbs like as *Melisa officinalis* (13.2 mg/g DW), *Taraxacum officinale* (12.6 mg/g DW), *Acorus calamus* (12.45 mg/g DW), *Echinacea purpurea* (15.1 mg/g DW), *Syzygium aromaticum* (8.96 mg/g DW) and *Salvia officinalis* (8.25 mg/g DW) pandan recorded lowest contents of TP [27,28].

**Table 2:** The concentrations of TP and some phenolic acids detected in pandan extracts from three different locations

	**Bachok**	**Klang**	**Pontian**
TP	6.72 ± 0.355^a^	5.07 ± 0.406^b^	4.88 ± 0.477^b^
Gallic acid	0.423 ± 0.052^a^	0.325 ± 0.041^a^	0.214 ± 0.019^b^
Cinnamic acid	0.084 ± 0.033^a^	0.033 ± 0.018^b^	ND
Ferulic acid	0.281 ± 0.037^a^	ND	ND

All analyses are the mean of triplicate measurements ± standard deviation. Results expressed in mg/g DW. Means not sharing a common letter were significantly different at P ≤ 0.05.

It is evident that the total phenolic content measured by the Folin–Ciocalteu method does not give a full picture of the quality or quantity of the phenolic compounds in the plant extracts [29]. In current research three phenolic acids including gallic acid, cinnamic acid and ferulic acid were identified in pandan extracts from three locations. The extract of pandan from Bachok exhibited highest value (0.423 ± 0.052 mg/g DW) of gallic acid compared to Klang (0.325 ± 0.041 mg/g DW) and Pontian (0.214 ± 0.019 mg/g DW) locations. In addition no significant difference was observed between Klang and Pontian locations for gallic acid production in pandan. As Table 2 shows, among the studied phenolic compounds cinamic acid was detected from Bachok and Klang locations. Bachok location represent high value (0.084 ± 0.033 mg/g DW) of cinnamic acid followed by Klang (0.033 ± 0.018 mg/g DW). Cinnamic acid was not detected in pandan extract from Pontian location. Ferulic acid was detected just in pandan extract of Bachok location with value of 0.281 ± 0.037 mg/g DW. Ferulic acid was shown to inhibit the photo peroxidation of linoleic acid which is potent fatty acid for decrease cancer risk, improve immune function, diabetes and heart disease prevention [23]. The most interesting finding was that ferulic acid just detected in pandan extracts from Bachok location. However, this result has not previously been described. Results imply that gallic acid is abundant pheolic acid between identified phenolics in pandan extracts.

### Antioxidant activity

#### Ferric reducing antioxidant potential (FRAP) assay

**Figure 2:**
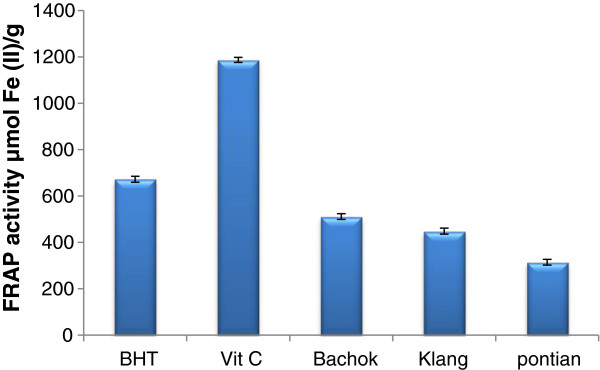
FRAP activity of pandan extracts collected from three different locations compared to the positive controls butylated hydroxytoluene (BHT) and vitamin C.

The FRAP assay depends upon the reduction of ferric tripyridyltriazine (Fe (III)-TPTZ) complex to the ferrous tripyridyltriazine (Fe (II)-TPTZ) by a reductant at low pH. The FRAP assay has been used widely to estimate the antioxidant component/power in dietary polyphenols [30]. As Figure 2 shows, the reducing power for the pandan extracts from three different locations was in the range of 511.2 (Bachok) to 314.8 μm of Fe (II)/g (Pontian). The FRAP values for the pandan extract in three locations were significantly lower than those of BHT (672.4 μmol Fe (II)/g) and Vit C (1186.55 μmol Fe (II)/g). In this study, we used the FRAP assay because it is quick and simple to measure the antioxidant capacity of purpose compounds and not only plants, wines, and animal tissues [31,32]. In general, antioxidant activity of flavonoids belong to the substitution pattern and structure of hydroxyl groups. In flavonoids chemical structure 30,40-orthodihydroxy in ring B and 4-carbonyl group in ring C are the fundamental requirement for effective radical scavenging. The presence of 3- and 5-OH groups, giving a catechol-like structure in ring C, is also essential for the antioxidant activity of flavonoid compounds [28]. Furthermore, the presence of the C2–C3 bond configured with a 4-keto arrangement is recognized to be responsible for electron delocalization from ring B and following that enhance the free radical scavenging activity. In the absence of the o-dihydroxy structure in ring B, a catechol structure in ring A can compensate for flavonoid antioxidant activity [33].

### 1,1-Diphenyl-2-picrylhydrazyl (DPPH) assay

**Figure 3:**
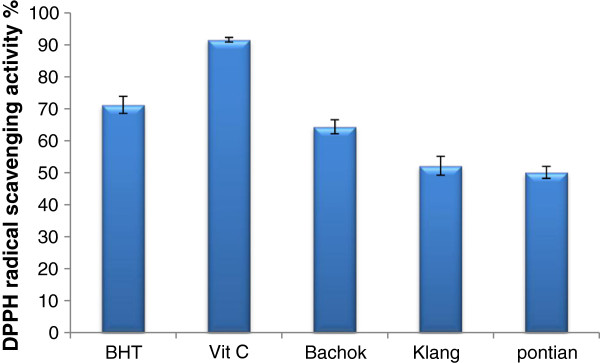
DPPH radical scavenging activity of pandan extracts collected from three different locations compared to the positive controls butylated hydroxytoluene (BHT) and vitamin C.

**Figure 4:**
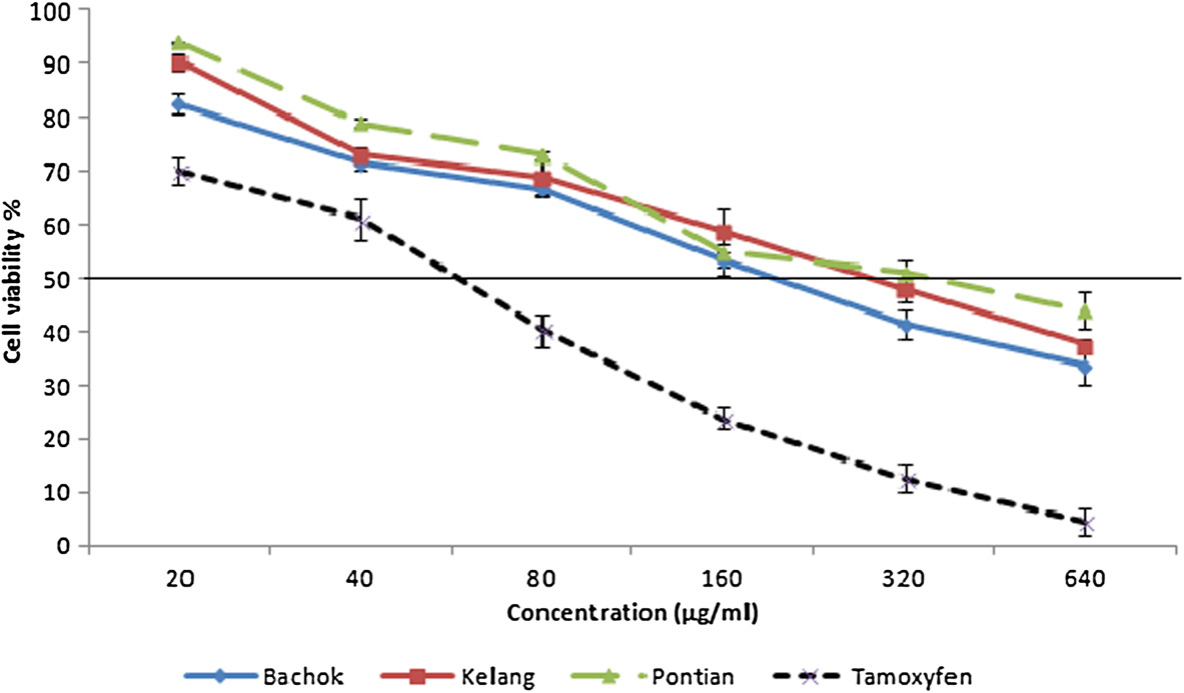
Dose-dependent anticancer of pandan extracts from 3 different locations (Bachok, Klang and Pontian) towards MCF-7 cell line as determined by the MTT assay.

The effect of antioxidants on DPPH scavenging is due to their hydrogen donating ability. Between studied locations Bachok exhibited highest value (64.27%) of DPPH activity followed by Klang (52.16%) and Pontian (50.10%), (Figure 3). In addition, significant difference was observed between three location for DPPH activity. The results of the current study showed that DPPH radical scavenging abilities of the extracts of pandan from three locations were less than those of butylated hydroxytoluene (BHT) (83.7%) and Vit C (92.3%) at 35 mg/mL. The IC_50_ (fifty percent free radical scavenging) value of pandan extract were 9.25, 11.6 and 12.5 mg/mL for Bachok, Klang and Pontian locations respectively. Sasikumar et al. [34] reported that the DPPH antioxidant activity in pandan extract were comparable with those obtained by Higher antioxidant potential was observed in both DPPH scavenging assay (EC = 48.350 ± 0.002 μg/mL) and reducing capacity (OD at 1000 μg/mL = 0.787) by the methanolic root extract than by the aqueous extract. Marinova et al. [35] but higher than that of Odukoya et al. [36]. When a comparison betweeen Figures 3 and 4 was made to appear the trend for ferric ions reducing activities of the pandan did not vary markedly from their DPPH free radical scavenging activities. Antioxidant compounds such as polyphenols may be more efficient reducing agents for ferric iron but some may not scavenge DPPH free radicals as efficiently due to steric hindrance [36].

Pulido et al. [37] reported that, in general, the ferric ion reducing ability of antioxidants correlates with the results from other methods used to estimate antioxidant capacity. Reducing DPPH radicals were also able to reduce ferric ions. Arnous et al. [38] reported a strong correlation between DPPH free radical scavenging ability and ferric ion reducing ability in wines.

### Anticancer activity

**Figure 5:**
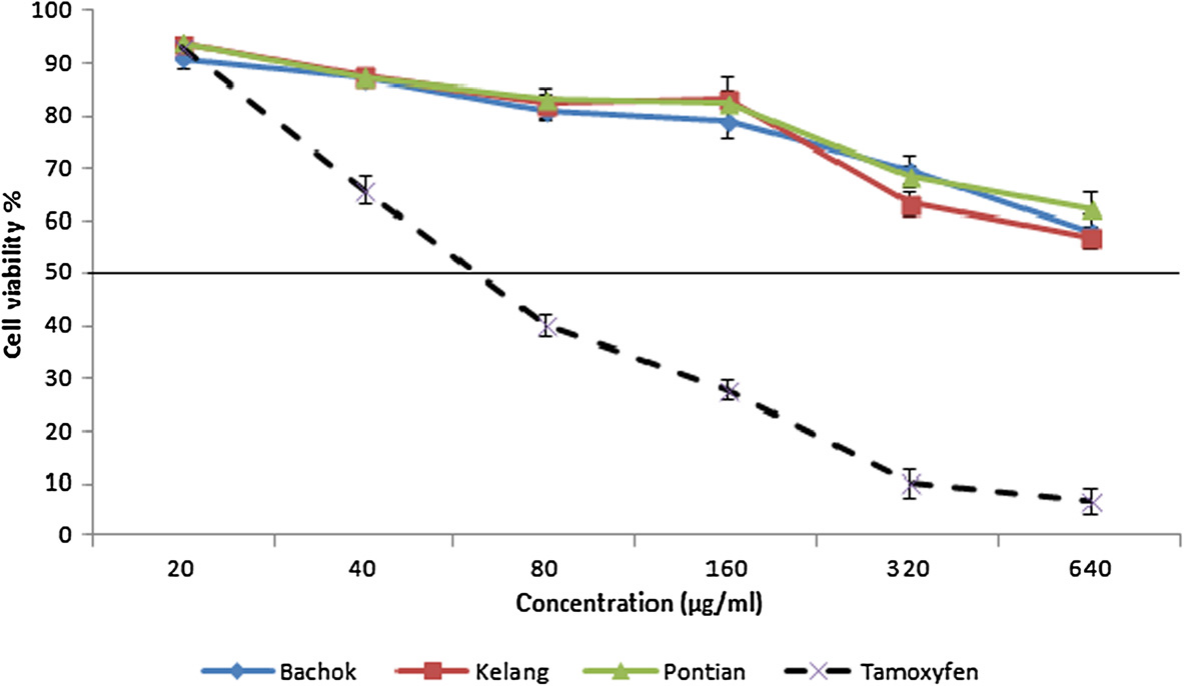
Effect of pandan extracts from three different locations (Bachok, Klang and Pontian) on normal cell (MCF-10A) viability.

The preliminary screening showed pandan extracts from 3 locations possessed anticancer promoting activity against MCF-7, with 78.3% 70.5% and 67.4 inhibition rate, respectively. Maximum MCF-7cell line inhibition was observed in pandan extract with values of 66.3% from Bachok location (Figure 4). MCF-7 cell lines treated with tamoxifen (positive control) showed 92.5% inhibition at the same concentration. According to obtained results, at a concentration of 160 μg/ml, however, pandan extracts from Bachok locations exhibited IC_50_ towards MCF-7 cells. The IC_50_ values of pandan extract from Bachok, Klang and Pontian locations against MCF-7 cells were 210.4, 285.6.6 and 334.2 μg/mL, respectively. Our finding is consistent with Zan et al. [39] who is reported IC_50_ value for *P. amaryllifolius* extract against breast cancer cell line obtain in >100 μg/mL. Flavonoids are among the best candidates for mediating the protective effect of diets rich in fruits and vegetables with respect to colorectal cancer [40]. Hence, flavonoid compounds could probably be responsible for the anticancer activity of curry leaf. Meanwhile, with the increase of extracts concentration, however, normal cell viability decreased in all extracts. In terms of toxicity to the normal cells (MCF-10A), *P. amaryllifolius* extracts from different locations were considered as not toxic as the IC_50_ values were greater than 640 μg/ml (Figure 5). In pandan extract from Bachok location at concentration of 210.4 μg/ml (IC_50_) the normal cell viability was recorded about 78%. *P. amaryllifolius* extract was found to display selective antiproliferative activity against non hormone dependent breast cancer cells [39]. In other study, ethanol extract of *P. amaryllifolius* induced apoptosis on hormone independent breast cancer cell line MDA-MB-231 [41]. In the current study the highest values of flavonoid compounds were detected in pandan extract from Bachok location. Meanwhile, the highest anticancer activity against MCF-7 cell lines has been observed with extracts of pandan from Bachok location. This suggests that high anticancer activity in pandan extract may be attributed to the high concentrations of potent anticancer components such as rutin, epicatechin, kaempferol and gallic acid. However, more research needs to be undertaken before the association between these flavonoids and anticancer activity in curry leaf is more clearly understood.

## Conclusion

In this study between three studied locations, the samples collected from the North (Bachok) which had a high content of phenolic acids (specially gallic acid) and flavonoids (specially catechin and kaempferol), had a very high antioxidant scavenging value in both FRAP and DPPH assay. Conversely, the samples collected from the Southern portion (Pontian) appeared to have the lowest content of TP, TF and antioxidant activity. Recently several plant derived natural compounds have been screened for their anticancer activity in order to identify putative compounds with novel structures or mechanism of action. In current study pandan extracts showed good potential of bioactive compounds such as catechin, gallic acid, kaempferol and naringin. It can be concluded that these bioactive compounds present in pandan extracts work synergistically in inhibiting proliferation of breast cancer cells and suggests that they may have potential for use as a natural additive in human diets. The wide ranges of the secondary metabolites content and antioxidant activities of pandan extracts could be due to many factors including locations, altitude, temperature, age of plant, climate and variation of plant variety. The ranges of phenolic acids and flavonoid content and antioxidant activity will be useful for standardization of pandan extracts for further pharmaceutical productions. These results also show the possibility of increasing the content of natural antioxidants by optimizing the growing conditions of pandan. More information on other bioactive component of pandan would help us to establish a greater degree of accuracy on this matter.

## Competing interests

The authors declare that they have no competing interests.

## Authors’ contributions

Experimental work was done by AG under the supervision of HJ (post doctoral project). The first draft of the paper was written by AG and reviewed by HJ. Both authors read and approved the final manuscript.

## Pre-publication history

The pre-publication history for this paper can be accessed here:

http://www.biomedcentral.com/1472-6882/13/341/prepub
